# Perceptions of Causes and Treatment of Mental Illness Among Traditional Health Practitioners in Johannesburg, South Africa

**DOI:** 10.1192/j.eurpsy.2023.748

**Published:** 2023-07-19

**Authors:** M. Galvin, L. Chiwaye, A. Moolla

**Affiliations:** 1 Psychiatry, Harvard University, Cambridge, United States; 2 University of the Witwatersrand, Johannesburg, South Africa; 3 Psychiatry, University of the Witwatersrand, Johannesburg, South Africa

## Abstract

**Introduction:**

Mental disorders are among the most poorly treated illnesses in sub-Saharan Africa. It is estimated that 70-80% of South Africans consult Traditional Health Practitioners (THPs) for the treatment of psychological ailments. Few studies have examined the perceptions of THPs regarding causes of mental illness and whilst we know little about their practices, THPs maintain a strong role in assessing and treating patients with mental illness.

**Objectives:**

This research aims to be among the first studies to identify perceived causes and treatment modalities for mental illness among THPs in Johannesburg, South Africa.

**Methods:**

Semi-structured in-depth interviews were conducted with 18 THPs in Johannesburg, South Africa between January and May, 2022. Interviews were transcribed and translated into English. Data was managed using NVivo 12 software and thematically analyzed.

**Results:**

THPs interviewed generally perceived mental illness to be of supernatural causation, either as a result of bewitchment, a calling for the patient to become a THP themselves, due to angry ancestors, or due to natural causes. THPs identified eight primary treatments that they use for treating mental illness. Among these were: throwing of bones (*tinhlolo*) to start communicating with ancestors, steaming (*ukufutha*) to start the cleansing process, sneezing (*umbhemiso*) to forcefully dispel the spirit causing the illness, vomiting (*phalaza*) and laxatives (*mahlabekufeni*) to remove the spirits poisoning the body as well as animal sacrifice to purge spirits and communicate with ancestors. This is all followed by cutting (*ukucaba*) which is the final part of treatment that ensures that the evil spirit cannot return.

**Conclusions:**

This study is among the first to examine the perceived causes and treatments for mental illness used by THPs in Johannesburg, South Africa. As the vast majority of South Africans continue to seek help for mental illness via THPs, it is important to understand what forms of care healers are providing to patients. Future research should continue to document ways in which THPs approach healthcare as well as investigate interventions that can foster collaboration between THPs and biomedical professionals.

**Disclosure of Interest:**

None Declared

